# Usability Evaluation Methods for Gesture-Based Games: A Systematic Review

**DOI:** 10.2196/games.5860

**Published:** 2016-10-04

**Authors:** Fernando Winckler Simor, Manoela Rogofski Brum, Jaison Dairon Ebertz Schmidt, Rafael Rieder, Ana Carolina Bertoletti De Marchi

**Affiliations:** ^1^ Universidade de Passo Fundo Passo Fundo, RS Brazil

**Keywords:** usability testing, evaluation, computer games, gestural input, usability evaluation, method, gesture-based games

## Abstract

**Background:**

Gestural interaction systems are increasingly being used, mainly in games, expanding the idea of entertainment and providing experiences with the purpose of promoting better physical and/or mental health. Therefore, it is necessary to establish mechanisms for evaluating the usability of these interfaces, which make gestures the basis of interaction, to achieve a balance between functionality and ease of use.

**Objective:**

This study aims to present the results of a systematic review focused on usability evaluation methods for gesture-based games, considering devices with motion-sensing capability. We considered the usability methods used, the common interface issues, and the strategies adopted to build good gesture-based games.

**Methods:**

The research was centered on four electronic databases: IEEE, Association for Computing Machinery (ACM), Springer, and Science Direct from September 4 to 21, 2015. Within 1427 studies evaluated, 10 matched the eligibility criteria. As a requirement, we considered studies about gesture-based games, Kinect and/or Wii as devices, and the use of a usability method to evaluate the user interface.

**Results:**

In the 10 studies found, there was no standardization in the methods because they considered diverse analysis variables. Heterogeneously, authors used different instruments to evaluate gesture-based interfaces and no default approach was proposed. Questionnaires were the most used instruments (70%, 7/10), followed by interviews (30%, 3/10), and observation and video recording (20%, 2/10). Moreover, 60% (6/10) of the studies used gesture-based serious games to evaluate the performance of elderly participants in rehabilitation tasks. This highlights the need for creating an evaluation protocol for older adults to provide a user-friendly interface according to the user’s age and limitations.

**Conclusions:**

Through this study, we conclude this field is in need of a usability evaluation method for serious games, especially games for older adults, and that the definition of a methodology and a test protocol may offer the user more comfort, welfare, and confidence.

## Introduction

Interactive systems can only be considered useful and practical if they have good usability. According to Karray et al [1], usability is the variety and the degree to which system features can be used efficiently so that the user can accomplish tasks effectively and intuitively. The balance between functionality and usability allows achieving the system effectiveness. Among the usability characteristics defined by Nielsen [2] are ease in performing basic tasks, efficiency when performing these tasks, facility by reusing resource, reestablishment of services when mistakes occur, and satisfaction with use.

Researchers in the area of human-computer interaction have been developing several usability evaluation methods in order to determine whether a system or interactive device is usable or not. According to Cockton [3], usability evaluation is essential to establish a relationship between the quality of an interactive system and interaction quality. The author mentions that when a usability evaluation shows that an application or device can be used, methods and metrics can determine the extent to which a system is easy and pleasant to use.

The constant development of usability evaluation mechanisms occurs due to the high supply of interactive systems on the market, constantly bringing to the user new ways of interacting. Gestural interactions are among the styles that have evolved in more recent years and used in largely in entertainment applications, such as virtual reality environments and games.

According to Morelli and Folmer [4], gesture-based games typically simulate real physical activities because they use whole-body gestures. These kinds of games are intuitive to play, they have successfully attracted users, and they provide different social forms of gaming—especially because they allow natural interaction and immersion. This interaction style is present through different motion-sensing input devices [5]. Microsoft Kinect and Nintendo Wii are examples of devices that use gestures as an interaction method. They apply unimodal and multimodal resources, meaning they can combine audio, video, and gestures to emulate interactive environments [1]. According to Karam and Schraefel [6], depending on the application type, it is possible to use more than one input device in the interaction base, which will all allow the same action.

Given the market demand for systems with gestural interaction, it is necessary to establish procedures to evaluate the usability of these interfaces in order to minimize interaction problems. In this perspective, Keskinen et al [7] proposed a method to evaluate user experience in interactive systems. Rautaray and Pandey [8] presented comparative studies that characterized gestural interaction elements and Maidi and Preda [9] organized how gestures could be evaluated. However, it is difficult to establish a consensus between these and other studies regarding what should or not be evaluated, especially when it comes to evaluating the usability of gestural interaction applied to games.

Different tools are being used now to assist the usability evaluation process: some present qualitative results and others quantitative, some focus on perception or acceptance, some consider physiological measures, and so on. The big gap is the lack of a validated approach that makes it possible to ensure consistent assessment results and increase its credibility. Still, usability patterns could be defined from this approach along time to use as benchmarks in evaluations.

In this sense, the aim of this study is to present a systematic review about the usability evaluation methods applied to games with gestural interaction, considering devices with motion-sensing capability. To reach this goal, specific objectives have been set: (1) identify and analyze techniques applied to usability analysis of games interfaces for gesture-based devices, (2) identify common problems found in gesture-based games interfaces, and (3) relate strategies and technologies that have been used to solve user interface problems in gesture-based games.

## Methods

This study is a systematic review, explicit and rigorous research that identifies, critically evaluates, and synthesizes relevant studies about a specific subject [10].

### Eligibility Criteria

The eligibility criteria to identify studies in the primary phase included were (1) games for gestural interaction devices, (2) Kinect and/or Wii as the gestural interaction device, and (3) description of a usability evaluation technique for analysis of user interfaces.

### Search Strategy for Primary Studies

The research was concentrated into four electronic databases: IEEE Xplore Digital Library, Association for Computing Machinery (ACM), Springer International Publisher Science, and Science Direct. This work collected studies published in English between September 4 and 21, 2015, not limited by date, and using the following expression: games AND usability AND evaluation AND (“Kinect” OR “Wii”).

### Flowchart of Identified Studies

**Figure 1:**
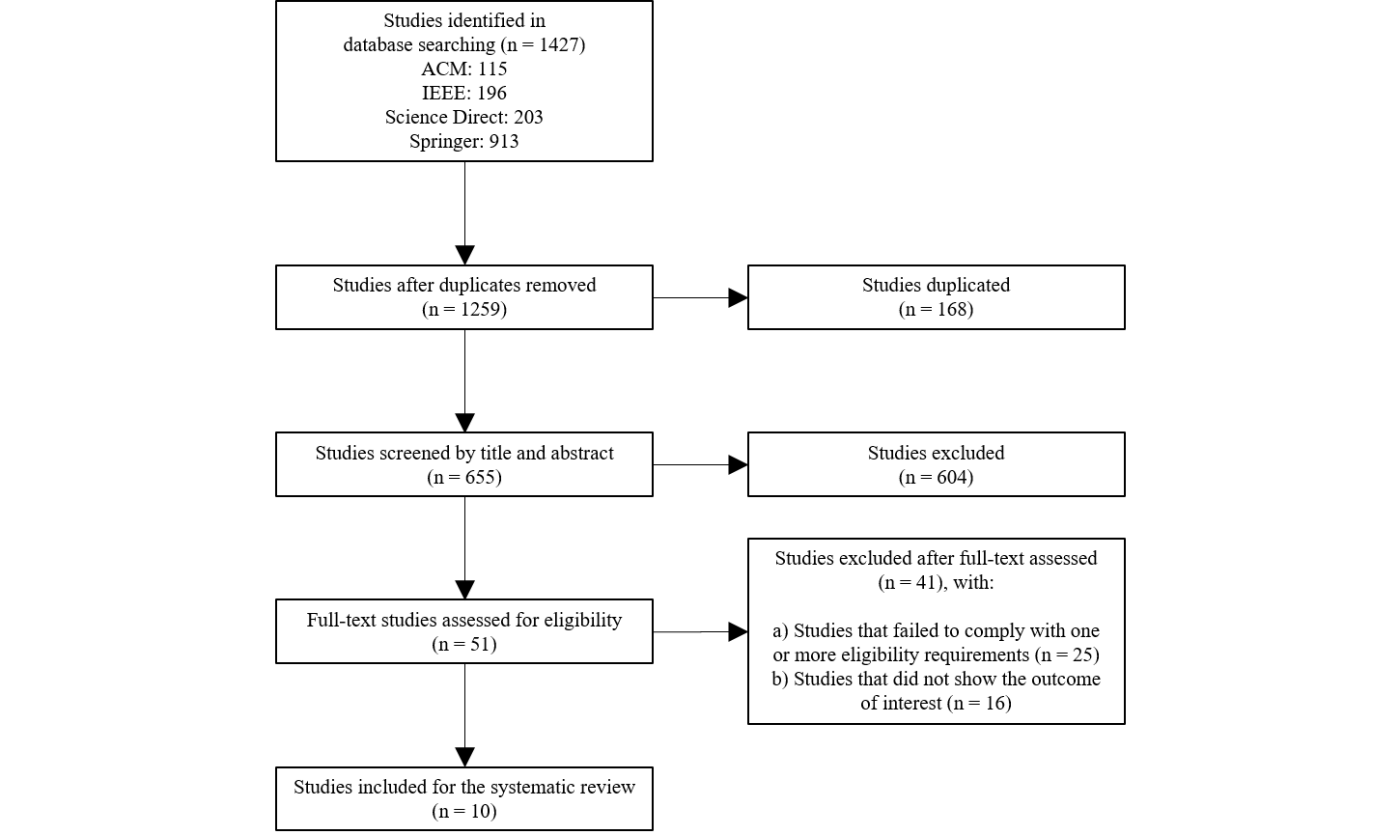
Flowchart of identified studies. ACM: Association for Computing Machinery.

Figure 1 shows the flowchart of studies identified by the search strategy used in this study. The selection process and reading of these materials involved at least two researchers.

## Results

Table 1 presents the 10 studies that met the eligibility criteria. These studies showed no standardization in their data or their methods, which made it impossible to conduct a statistical analysis.

Each study will be presented considering the following items:

1. Evaluation aim: approach and focus of each study;

2. Gesture-based devices used: equipment used during the experiments;

3. Evaluator’s profile: professionals and researchers team;

4. Profile and number of participants: characterize the sample;

5. Evaluation method and its application steps: the author’s methodology and how it was applied;

6. User’s tasks: experiment tasks;

7. Type of interface (2D or 3D) and software used: the applications used during the experiments;

8. Time required for the user experience and evaluation: the period for each user evaluation; and

9. Results: the analysis and conclusions of the authors of each study.

### Komlódi Et Al

Komlódi et al [11] aimed to test the Wiimote device in basic navigation, object manipulation, and menu gestural interaction tasks in a virtual environment. The users perform pointing and turning gestures using their hands and the Wii controllers. During the interaction process, they also pushed the buttons of the controllers to operate different navigation modes.

Researchers from the University of Maryland in the United States and the Budapest University of Technology and Economics in Hungary conducted the evaluation. A group of 14 Hungarian undergraduate students (seven men and seven women) participated in the evaluation; the mean age of participants was 24 years. Half of these users had some experience with computer game simulators. Four had used the Wiimote previously, but none had significant experience.

Although this study was not a game, it was included because, in the demographic questionnaire, the authors considered the user’s previous game experience important. According to Bowman et al [21], this has a strong impact on the ability to interact with virtual environments. Moreover, virtual environments can simulate or represent a game in 3D space. In this study specifically, one of the user tasks involved the handling of dominoes using the Wiimote.

During the pilot test, three participants performed the tasks in a virtual environment and answered questionnaires. The procedure had several changes throughout the testing period: extension of the training period, review of tasks, and changes in the interview questions.

The study combined qualitative and quantitative methods to explore the utility of interaction methods in a 3D environment. After a brief training session, 14 participants executed two immersive tasks in three pilot tests. A video camera recorded the entire process and the researchers interviewed participants about their experience immediately after the interaction.

After the orientation and training session, participants read instructions for tasks on a poster. This poster displayed the following guidance: (1) walk around the room, find the dominoes, stack the dominoes one on top of another, and dismantle them; and (2) use the KUKA robot to move the gray black balls on the table and, when finished, tell the session coordinator.

Participants answered a questionnaire containing 19 questions about their experience using the game. Some questions were issues, such as ease of interaction, first impressions, reactions during use, satisfaction when using, and suggestions for future game improvements. Only two questions used a scale of 0 to 100; the others were dissertated.

In addition to this questionnaire, participants were also requested to complete a sociodemographic questionnaire and the Myers-Briggs Type Indicator (MBTI) personality type questionnaire. This questionnaire identifies users’ characteristics of extroversion/introversion, sensing/intuition, thinking/feeling, and judgment/perception. Participants also performed the VZ-2 paper folding test to measure cognitive ability of spatial visualization, which can influence the user’s ability to navigate in 3D space and manipulate objects in space, and the Reading the Mind in the Eyes Test.

For effective usability studies, the authors found the need for more training time and practice with games as well as several sessions of interaction involving experts and novices with games and devices. In addition, reducing the memory load for users by including tasks and feedback functions, as well as help in the environment also improves the game usability.

### Legouverneur Et Al

Legouverneur et al [12] aimed to conduct a usability study of two sports games for the Wii. The purpose was to determine whether the elderly with cognitive impairment could learn to play and control their movements with the wireless controller (Wiimote). A secondary objective was to examine how specific neuropsychological deficits may modulate the game usability.

Broca Hospital professionals from Paris, France, conducted the evaluation with two groups of users recruited from a health care center. The first group consisted of elderly people with mild-to-moderate Alzheimer disease as the diagnostic criteria. The second group consisted of elderly people with mild cognitive impairment. A third group consisted of healthy elderly individuals. All users were aged between 75 and 90 years.

The test protocol included an introductory session and four test sessions, with mean duration of 1 hour per week. The introductory session included a neuropsychological evaluation. During this session, participants also created their own avatar as a way to learn to use the Wiimote. In the test sessions, the participants interacted with two bowling games and two tennis games alternately. Each test session had two cameras to record the game screen and the player simultaneously.

The use of the Wiimote was to mimic the actions of swinging a racket (tennis) or rolling a ball down an alley (bowling). According to the authors, the movement performed by each user was analyzed considering the approach of games (no specific gesture was evaluated).

In addition to the game console and cameras, other equipment involved in the experiment was a 46-inch plasma TV and behavioral analysis software. In order to collect performance and behavior data, the authors used the video recordings. A questionnaire using a five-point Likert scale evaluated user preferences. The authors used the questionnaire at the end of the first test session and after session 4 to evaluate whether familiarity with the games influenced user preferences.

The results showed that all participants, regardless of their cognitive status, could use the wireless controller and learn to play both games. A positive experiment result, according to the authors, was the improvement of skills with games throughout the sessions on performance measures observed for most participants. The study also confirmed the importance of usability testing with end users before introducing traditional technology to older adults who have cognitive dysfunction. Multiple sessions allowed users with cognitive impairment to be comfortable with technological devices in order to learn how to use them and have a positive experience with them. This experience also confirmed the role of motivation and a socially supportive environment on how a person learns to use new technologies.

### Francese Et Al

In the Francese et al study [13], the aim was to evaluate two games developed for 3D interaction with the use of navigation maps from Bing Maps. The games used were Wing for the Wii and King for Kinect. The main objective was to evaluate the gestural interaction by controlling user navigation in Bing Maps through the devices previously mentioned.

With the Wiimote, the gestures were inspired by the motorcycle metaphor: roll/rotation of the Wiimote acts as a motorcycle throttle command connected to navigation forward and backward movements; the turning gestures resemble the turning handlebar of an imaginary motorcycle. With the Nunchuk, the airplane cloche metaphor was used to control altitude: its tilting direction determined the vertical variations of the navigation.

Using Kinect, the bird (or airplane) metaphor was used, with natural gestures associated to the various commands. The idea was to mimic the bird’s wing movements, when possible, with arm gestures. For example, the user would move their aligned arms downward to the left as the bird or airplane did onscreen. A virtual paper plane was presented to the user to give a feedback about their movement.

The evaluation process involved 24 volunteers (16 men and 8 women), who were staff and students from the University of Salerno in Salerno, Italy. Ages ranged between 18 and 41 years, with a mean of 24 years.

Before beginning the experiment, the skills of participants in the games were evaluated. Eight participants mentioned they played at least once a week, three played Wii, and only two played Xbox and Kinect. Each participant answered 12 questions, using a seven-point Likert scale, and three factors were evaluated: involvement, distraction, and control. The study was performed in a research laboratory of the University of Salerno.

For the experiment, participants were quickly introduced to gestural interfaces and performed two navigation tasks. After being instructed on how to use both games, Wing and King, users were asked to navigate in two geographical routes involving well-known Italian cities: MAR (Cagliari-Naples-Palermo) and TERRENO (Genoa-Rome-Venice).

Both tasks were compatible in terms of distance and difficulty in locating the destination cities. In order to avoid bias in evaluation tasks, the approach defined two user groups in which each member of the same group started the experiment with the same system. After each task, all participants filled-in After-Scenario Questionnaires (ASQ) to evaluate the time spent, the ease of completion, and the adequacy of support information.

Authors used the Computer System Usability Questionnaire (CSUQ), consisting of 19 questions, to evaluate user satisfaction with the 3D maps game for four factors: general evaluation, system utility, information quality, and interface quality. According to the authors, evaluation results conducted through questionnaires confirmed that, if the interface is more natural, the user will be as satisfied and engaged in the navigation experience.

**Table 1:** Studies included in the systematic review.

Study ID	Paper title	Device
Komlódi et al [11]	Empirical Usability Evaluation of the Wii Controller As an Input Device for the VirCA Immersive Virtual Space	Wii
Legouverneur et al [12]	Wii Sports, a Usability Study with MCI and Alzheimer’s Patients	Wii
Francese et al [13]	Wiimote and Kinect: Gestural User Interfaces Add a Natural Third Dimension to HCI	Wii and Kinect
Norouzi-Gheidari et al [14]	Interactive Virtual Reality Game-Based Rehabilitation for Stroke Patients	Kinect
Liu et al [15]	An Approach of Indoor Exercise: Kinect-Based Video Game for Elderly People	Kinect
Shin et al [16]	A Task-Specific Interactive Game-Based Virtual Reality Rehabilitation System for Patients with Stroke: a Usability Test and Two Clinical Experiments	Kinect
Fang et al [17]	Interactive Physical Games: Improving Balance in Older Adults	Kinect
Harrington et al [18]	Assessing Older Adults’ Usability Challenges Using Kinect-Based Exergames	Kinect
Nakai et al [19]	Investigating the Effects of Motion-Based Kinect Game System on User Cognition	Kinect
Sheu et al [20]	User-Centered Design of Interactive Gesture-Based Fitness Video Game for Elderly	Kinect

### Norouzi-Gheidari Et Al

Norouzi-Gheidari et al [14] developed a study to use a research protocol to validate a virtual reality system for rehabilitation. This system used five games for the motor recovery of the upper limb in stroke survivors. The gestural interaction device used was the Kinect. The evaluation involved eight health professionals, with at least 1 year of experience in neurological work, who evaluated 24 stroke patients with different skill levels.

The games’ activities used arm movements (unilateral and bilateral) and torso control in the sitting position. For this evaluation, the active range of motion of the arm was only considered within the context of reaching for each patient to determine target placement for the activities.

For the experiment, the patients were divided into four groups of six patients based on level of motor ability. Each patient participated in three 20-minute game sessions of over 10 days. During each session, patients should sit in a chair in front of the Kinect camera at a fixed distance of 1.5 meters as per device specifications. The calibration at this distance was important because the active range of arm movement within the game’s reach space determined the target position for the activities.

The patient interacted with all five games at least once during the 20-minute session. The doctor defined the level of difficulty of each activity (eg, required speed, destination number, repetitions), which could be adjusted during the session.

After interacting with each game, the patient had access to a score of correct answers (according to their level of performance) as a way to encourage him/her to continue. At the end of each session, the doctor had access to a global performance report. In the final session, doctors and patients should complete a questionnaire based on the Technology Acceptance Model (TAM) of medical information to assess their opinions about the game system. Additionally, the Fugl-Meier Assessment of sensorimotor recovery after stroke test evaluated the upper limbs. For each patient, session, and activity, they measured the success rate, the performance in tests of success (medium speed and precision), and highest difficulty level reached within the session.

However, this article showed only preliminary results, specifically compiling and identifying the advantages and limitations perceived by clinicians and patients with stroke in rehab games. Success rates, performance scores, and difficulty levels have not been studied yet.

### Liu Et Al

Liu et al [15] evaluated the usability of a game they had developed, which used the Kinect device. The game allowed users to accumulate points and stimulated competition among friends. The interaction tasks were to select bubbles that fell from the top of the screen area.

The authors randomly chose six volunteers in a park (two men and four women), who were aged between 50 and 88 years.

The players were able to move one of their hands to select any button options to configure the game. During the interaction process, hands were used freely to select the game objects.

The evaluation experiment consisted of three stages: a brief introduction to the game, testing activity (use the game), and a follow-up interview to collect their comments. First, the evaluator presented the game and the user could ask questions. After that, the user played the game prototype for 1 minute then answered a list of open questions and provided any suggestions for the game design. The activities performed by the users when using the game consisted of indoor exercises, like jump, hit, and grab onto it.

As for results, the authors emphasized some issues. First, the video game presents less danger to the player. Second, it offers more entertainment and, therefore, motivation. The mechanism of obtaining points and prizes by exercising helped the players to increase the amount of exercise unconsciously. The event of winning served as a strong motivator, while also helping to maintain long-term exercise habits for the elderly. Third, through the online gaming platform, players could still have fun together with their friends as if being together somewhere. All current devices work individually and keep the elderly away from their companions, which could have a negative impact on their social life. However, online platforms offer access to players to share their scores and comments with their friends. This not only increased the game’s entertainment, but also added competition among friends, helping with motivation and having a positive effect during the exercises. Although all users showed interest in the game and provided a good evaluation, the evaluation questionnaires showed some directions to consider improving the game. The game should have different difficulty levels and diverse tasks. Game messages should provide clearer instructions and not just information. In addition, it must provide kinds of exercises that can help exercise different body parts, yet must be simple for easy learning and understanding by the elderly.

### Shin Et Al

Shin et al [16] aimed to combine gestural rehabilitation exercises with game elements using PrimeSense technology, which are 3D-depth camera sensor chips part of Microsoft’s Kinect motion-sensing system. Researchers at the University of Hanyang in Seoul, South Korea, conducted the evaluation. The organization of two user groups was as follows: stroke patients and health professionals (occupational therapists and physiatrists) who were involved in the software design of RehabMaster, a game-based virtual reality rehabilitation system developed by the authors. Two clinical studies were performed; the first with seven patients and the second with 16 patients, all diagnosed with stroke. The first was an observational study in which seven patients with chronic stroke received the RehabMaster intervention for 30 minutes per day for 2 weeks. The second was a randomized controlled study of 16 patients with acute or subacute stroke, who received 10 sessions of conventional occupational therapy and plus 20 minutes of the RehabMaster intervention.

The authors evaluated patients’ routine tasks individually and focal group studies were performed once a week for approximately 6 months. The software categorized user feedback during the development process.

Regarding gestures and motions, the interventions aimed to stimulate patients through tasks using arm and trunk movements. The motions were intended to promote incremental improvement in range of motion and endurance, strength, and deviation from synergistic motion patterns. RehabMaster provided games to train the patient’s forearm movement and eye-hand coordination; upper extremity control, endurance, speed, accuracy, and range of motion; and to increase the control, speed, and accuracy of extremity control and trunk movements.

The same users also participated in the usability study later on. The objective was to evaluate RehabMaster from the perspective of each group. Meetings held with stroke patients took 20 minutes at regular intervals, twice a week for two weeks, under supervision of occupational therapists and physiatrists. Each of the three groups answered a different questionnaire using a five-point Likert scale, so the authors could collect diverse viewpoints. The Fugl-Meyer Assessment and the modified Barthel Index also were used during the evaluation.

Patient involvement was a key point of the RehabMaster intervention. With the stroke patients, the authors wanted to evaluate RehabMaster’s ability to provide strong motivation, pleasure, and an optimal flow experience. With the secondary user group (occupational therapists and physiatrists), they wanted to assess the usability of RehabMaster for improving upper limb function and the ability to provide adequate challenge levels for all different patients in the stroke group.

To diagnose if the game provided stroke patients with a desirable rehabilitation level, the study considered three factors in their game experience: attention maintenance, ability, and motivation. These factors were identified through six questions asked of participants. Generally, it found that participants had serious attention and a pleasant experience (immersion), even considering the users’ motor limitations.

Tests showed the viability of using RehabMaster in stroke patients with different levels of severity within a safe virtual environment. However, their results were inconsistent due to the different experimental protocols using different intervention times in both experiments.

The authors emphasized the need for a new study. One reason was because cognitive function, motivation, and depression, which are common in stroke patients, were not considered. Another factor was that the usability evaluation did not compare the perspectives of each group.

### Fang Et Al

Fang et al [17] developed an interactive prototype motion-based game called Evergreen Fitness System (EFS), in order to train balance in older adults. Health care experts carefully selected the exercises for the users.

The EFS recognizes body gestures and body motions using Kinect. Gestures were used to select the menu (hand movements), whereas body motions were required as part of the exercises available on the game system. Because the goal was to improve senior’s balance performance, the focus of the exercises was on lower body strength. Tasks developed consisted of specific exercises for balance training and strengthening of the lower limbs. Six exercises designed for improving balance explored knee marching, side hip raise, lunges, partial squats, wide squats, and standing knee flexion.

Thirteen participants were involved in the study (2 men and 11 women) aged between 60 and 80 years. The study used six exercises, specifically designed by experts to increase lower body strength in older adults, and integrated the elderly in games. Before the test, users were asked to complete a Physical Activity Enjoyment Scale (PAES) questionnaire to evaluate if they were physically capable of performing the test. After the exercises, participants answered a second questionnaire (Physical Activity Readiness Questionnaire, PARQ) that measured the degree of pleasure performing the activity [22].

This study showed that elderly participants approved the exercises based on games, culminating in a positive experience with the EFS. They provided feedback on improving the system design, on the appropriateness of the six exercises, system operation, game design, and demonstrated intention of using the game. It also verified the system should include a navigation requiring less learning, corrective feedback, and warnings while idle.

### Harrington Et Al

Harrington et al [18] stated that few studies had examined the usability challenges faced by the elderly using exergames. Thus, it is necessary to identify these challenges and how they translate into guidelines to provide user-friendly exergames for seniors. The objective of this study was to identify the challenges of usability based on Kinect exergames for seniors. Particularly, it aimed to identify which were the most difficult assimilation aspects for the elderly. To do so, 10 people aged between 60 and 69 years (five male and five female) and 10 people (five male and five female) aged between 70 and 79 years, recruited from Georgia Institute of Technology, participated in the study. Pretrials ensured that participants would be able to perform the expected actions; none had experience with the Microsoft Xbox 360 or any device that used Kinect.

The proposed activities were two games that encouraged physical activity: “Body and Brain Connection” and “Your Shape Fitness Evolved.” Both games used body motion, providing different activities as participants used their hands or feet to select objects, used their arms in balance challenges, and did torso exercises. Hand gestures were used to select a particular activity in each game, without any restriction.

The evaluation was developed as follows: before participating, each participant completed a health questionnaire, a demographic questionnaire, a technology experience survey, and a game experience questionnaire. Health and demographic questionnaires evaluated the health of the participants and collected basic information, including age, sex, race, education, and limitations (vision, hearing, or mobility). The technology experience questionnaire evaluated the use and familiarity of participants with various technologies. The game experience questionnaire [23] evaluated the participants’ levels of familiarity with games and their playing habits.

Additional questionnaires were filled out after each individual session to evaluate user satisfaction and performance. A questionnaire with five items measured satisfaction with the motion controls and gestures for navigation. A seven-item questionnaire assessed satisfaction with the activity developed in the program. Both questionnaires used a scale ranging from 1 (strongly disagree) to 7 (strongly agree). The game experience questionnaire was adapted from Boot et al [23]; information about the other questionnaires was not detailed.

After completing the questionnaires, participants interacted with the Kinect device, including training and definition of the participant’s starting position. Test sessions began with researchers giving details of what would be required from the participant. Researchers informed the participants that they could stop the test at any time if they felt they could not complete an activity. After that, an interview assessed participant behaviors and opinions about the programs and the experience. During the interviews, the participants described what they liked and disliked about each program and their line of thought. In addition, participants also answered if they used some kind of help or additional instruction throughout the program. The purpose of these interviews was to determine what made participants feel more frustrated and what types of assistance would be most beneficial.

After completing both exergame programs, each participant completed three questionnaires. The first questionnaire evaluated usability and included the following propositions measured on a Likert scale ranging from 1 (strongly disagree) to 7 (strongly agree): clear and understandable system interaction, useful system for daily life, daily use of game to make one more physically active, and it improves well-being. The second questionnaire assessed the ease of use including the following items measured on the same Likert scale: easy to use, flexibility to interact, increase of skill, clear tasks, and learn to use. The first and second questionnaires were adapted from Davis et al [24]. The third questionnaire, the System Usability Scale (SUS), was adapted from Brooke [25], and was used to give a global view of subjective assessments of usability. Sessions lasted between 1.5 and 3 hours for each participant.

The study showed that older people realize the benefits of exergames, believing it to be a useful means to exercise. Regarding ease of use, the responses were diverse. Most participants in the 60 to 69 year group agreed that the interface was friendly, whereas most in the 70 to 79 year group disagreed with ease of use.

### Nakai Et Al

Nakai et al [19] conducted a study to evaluate the usability of a game using the Kansei engineering method or “feelings engineering.” Kansei engineering is a method to develop or improve products and services by translating the customer’s psychological feelings and needs into the domain of product design. It explores the emotions between a user and a computer system. The study involved 12 users who performed the tasks and system usability testing. They had 10 minutes to play different levels in a game prototype called “The Glider.”

In this game, the user controls a virtual glider using body motions and rotations, such as front-back movements to change speed and pitch axis, left-right movements to change direction and roll axis, and torso rotations to control yaw axis. A Kinect device was used to capture the movements of users.

This approach divided the evaluation into five steps as follows: questionnaire, behavioral observation, speech watching, game testing, and analysis. First, the authors developed a gaming environment, which was tested preliminarily by three participants. There were two game preparation sessions. Then, a pretest questionnaire collected basic information about the topics. After the observations, they used a posttest questionnaire to collect participants’ impressions.

During these observations, speech and behavioral data from participants were collected based on the think-aloud method. In this method, players are invited to express aloud what they are thinking, doing, and feeling. Therefore, the researcher must take care to explain the experiment purpose to participants, making it clear that is not to test the player’s playing skills, but the product itself. It is important to clarify the aim so that users know what is being analyzed so the quality of the experiment is guaranteed. Users performed the task and reported their feelings about the product whenever they failed to complete any of the tasks. In addition, users reported their impressions and thoughts; the analysis of these data depended on the task observation, recorded in a time sequence.

The results demonstrated that the first four levels had good playability and the necessity for players to receive the largest possible amount of information about the game. Motivation was the key point in the game because while they were motivated the game flow looked promising: the game attracted the players’ attention and players showed eagerness to learn new things.

### Sheu Et Al

Sheu et al [20] aimed to address issues on how to design a gesture-based system that allows older people to play in a secure, convenient, and enjoyable way. This study used two gesture-based games (EG I and EG II) developed by researchers for Kinect. The EG II is an optimized version of the EG I based on the feedback obtained from usability tests performed in the first game.

The exercises used gestures for selections (eg, swing right arm to the right to make cursor move one step to the right and use left arm for moving cursor to the left).

Seven users participated in the experiment (four men and three women), who were aged between 60 and 77 years. The selection criteria for the participants were not detailed. The test had three stages: (1) pretest questionnaires and basic living information for user selection; (2) procedure introduction (game), signing the consent form, and using game for the test procedures (tasks); and (3) posttest questionnaires and interview. Tasks performed consisted of selecting operations in the program interface.

Users were able to complete all tasks. On average, users performed the tasks more quickly on the EG II interface, suggesting that EG II was more usable than EG I. Furthermore, the subjective score for EG II was higher than for EG I. For task selection, it was suggested that, in terms of effectiveness and efficiency, vertical selection works better than horizontal selection because moving the right arm to the right to move the cursor to the right and the left arm to the left to move the cursor to the left can be exhausting. If this movement is vertically oriented, the system interaction becomes less tiring.

To compile the results from all studies, Tables 2 and 3 present the main characteristics of each study and their results to correlate the differences and provide support for the Discussion section.

**Table 2:** Summarization of the studies included for the systematic review.

Study ID and publication year	Evaluation methods	Focus and devices	Evaluation stages	Participants
Komlódi et al [11] (2011)	Sociodemographic and health questionnaire, observation, video recording, MBTI^a^, Folding Test, Eyes Test	Test device for navigation and task manipulation using gestures (Wii)	(1) Questionnaires and tests; (2) verbal guidance and reading; (3) tasks in a virtual environment; (4) evaluation	N=14 (7 men, 7 women); age: mean 24 years
Legouverneur et al [12] (2011)	Author’s questionnaire, video recording	Conduct a usability study for 2 sports games (Wii)	(1) Neuropsychological evaluation; (2) sessions tests	N=undefined; age: range 75-90 years
Francese et al [13] (2012)	ASQ^b^, CSUQ^c^, Presence Questionnaire	Evaluate two 3D interaction games in navigation tasks (Kinect and Wii)	(1) Questionnaire; (2) instructions; (3) tests; (4) ASQ and CSUQ questionnaires	N=24 (16 men, 8 women); age: range 18-41 years
Norouzi-Gheidari et al [14] (2013)	User performance report, author’s questionnaire, TAM, Fugl-Meyer	Using a protocol for evaluating a virtual reality system as motor rehabilitation tool of upper limb (Kinect)	(1) System interaction; (2) questionnaires and evaluation	N=24 with stroke; age: undefined
Liu et al [15] (2014)	Interview	Evaluate a game usability for select objects with top-down movements (Kinect)	(1) Game introduction; (2) game activities; (3) interview	N=6 (2 men, 4 women); age: range 50-88 years
Shin et al [16] (2014)	Author’s questionnaire, Observation, Fugl-Meyer, Barthel	Combining rehabilitation exercises with game elements (Kinect)	Different experimental protocols	Group 1: n=7, group 2: n=16; age: undefined
Fang et al [17] (2015)	Interview, PARQ^e^, PAES^f^	Check the user’s experience; train the equilibrium in elderly with upper limb (Kinect)	(1) Physical evaluation; (2) exercises; (3) satisfaction evaluation	N=13 (2 men, 11 women); age: range 60-80 years
Harrington et al [18] (2015)	Sociodemographic and health questionnaire, author’s questionnaire, Interview, technology experience and videogame experience questionnaires, TAM, SUS^g^	Identify usability challenges based on exergames for seniors (Kinect)	(1) Questionnaires; (2) training; (3) test; (4) interview; (5) satisfaction questionnaires and usability	Group 1: n=10 (5 men, 5 women), age: range 60-69 years; group 2: n=10 (5 men, 5 women), age: range 70-79 years
Nakai et al [19] (2015)	Sociodemographic and health questionnaires, author’s questionnaire, video recording, think-aloud protocol	Evaluate a game usability using evaluation methods based on Kansei Engineering (Kinect)	(1) Questionnaire; (2) behavioral observation; (3) think-aloud; (4) game test; (5) analysis	N=12; age: “seniors”
Sheu et al [20] (2015)	Sociodemographic and health questionnaire, Interview, PARQ, PAES, SUS	To list design issues of a gesture-based system that allows seniors to interact naturally in selection tasks (Kinect)	(1) Questionnaires; (2) game introduction; (3) test procedure; (4) questionnaires posttest; (5) interview	N=7 (4 men, 3 women); age: range 60-77 years

**Table 3:** Summarization of results of included studies.

Study ID and publication year	Results
Komlódi et al [11] (2011)	For effective usability studies, it is necessary to provide more training time and practice with games. In addition, reducing users memory load, including tasks and feedback functions, and environment helps also improve games usability.
Legouverneur et al [12] (2011)	All participants, regardless of their cognitive status, could use the wireless controller and learn to play both games.
Francese et al [13] (2012)	If the interface is more natural, the user will be as satisfied and engaged in the navigation experience as you want.
Norouzi-Gheidari et al [14] (2013)	This review presented preliminary results. It aimed to compile and identify benefits and limitations perceived by clinicians and patients with stroke in rehab games.
Liu et al [15] (2014)	The game offers more entertainment and less physical risks than physical activity, and may motivate seniors to increase the practice of exercises to get more points.
Shin et al [16] (2014)	It is necessary to define a standard assessment protocol and a time of intervention in order to evaluate the usability of the game.
Fang et al [17] (2015)	Seniors like exercises based on games and showed a positive experience using EFS.
Harrington et al [18] (2015)	Regarding ease of use, most participants in 60-69 year group agreed that the interface was friendly, whereas most in 70-79 year group disagreed with ease of use.
Nakai et al [19] (2015)	It is necessary to provide a game training session and initial guidance to the participants. The motivation proved to be a key point.
Sheu et al [20] (2015)	The way of selecting elements in the program interface must be vertically.

^a^MBTI: Myers-Briggs Type Indicator.

^b^ASQ: After-Scenario Questionnaire.

^c^CSUQ: Computer System Usability Questionnaire.

^d^TAM: Technology Acceptance Model.

^e^PARQ: Physical Activity Readiness Questionnaire.

^e^PAES: Physical Activity Enjoyment Scale.

^f^SUS: System Usability Scale.

## Discussion

### Overview of Selected Studies

Of the 10 selected studies, seven used the Kinect device for interaction, two used the Wii device, and one used both devices. From this observation, it is possible to propose a study about the reason for this difference because the interaction device may interfere positively or negatively in a usability evaluation. This research could evaluate, for example, if there is really a usability difference between both devices or if the increased use of Kinect is due to its popularity and its complete controller-free gaming experience.

In relation to usability evaluation, nine studies used games in their experiments. Of these, six studies were directed toward the elderly (60%), showing that there are several efforts in serious games for this population. Thus, it is evident there is a need for creating a usability evaluation protocol for serious games for seniors, capable of generating qualitative and quantitative results, because there were no standard serious game evaluation testing protocols found in this research. This trend is supported by the requirements needed to adapt the interface according to age including, for example, the sensitivity of effort and having enough time to do the tasks. In addition, there can be evaluated potential differences in serious game evaluations of 2D or 3D gaming interfaces for the elderly.

Of the nine studies that used games to assess usability, eight studies (89%) used games developed by researchers and only one [12] used a commercial game, in this case for the Wii. This result promotes questions about what caused this situation. It can evaluated as there are no games on the market that meet the objectives of the proposed studies or as the existing games would not be adequate for testing for some reason.

According to Harrington et al [18], inside the elderly population there is further fragmentation that results in groups with special needs for good interface usability. In their study, the majority of participants in the 60 to 69 year group agreed that the interface was friendly, whereas most of the 70 to 79 year group disagreed with ease of use. It is necessary to identify these challenges and apply them into the development process of recommendations for the project in order to provide user-friendly systems to the elderly population and its subgroups.

On the other hand, the study from Legouverneur et al [12] showed that all participants, independently of their cognitive status, were able to use the wireless controller and learn to play both proposed games. They also argued that seniors could improve their game skills throughout the sessions based on their collected performance measures. Yet, several usability sessions allowed users with cognitive impairment to become familiar with technological devices and learn how to use them and have a positive experience.

Nakai et al [19] determined that it is necessary that players receive guidance about the game. This can be an alternative for the same prototype to be applied to many elderly groups even if they have different characteristics.

Most of the studies identified motivation as a major incentive for the elderly to use games to practice physical activities. Zhao et al [26] confirmed this condition, showing that physical exercises are the main intervention instrument in the preventive health and rehabilitation area. In this context, Cary et al [27] and Göbel et al [28] emphasized that serious games are alternative tools and aids for disease prevention. They can stimulate the practice of beneficial activities to human body and increase the patient’s interest for his treatment, which is often slow and painful [29]. Another factor that can encourage motivation in users is the use of movement track devices; the more natural the interaction process, the more the user is satisfied and motivated to explore the game resources [13].

Concerning evaluations, questionnaires were the most used instruments (70%, 7/10 studies), followed by interviews (30%, 3/10 studies) and observation and video recording (20%, 2/10 studies). There was also use of think-aloud methods and the Folding Test, but both were applied only in one study.

All studies that used questionnaires were evaluating the interface exclusively. Only two studies used sociodemographic questionnaires [11,18]. All approaches used a questionnaire to evaluate user experiences with natural interaction devices. Four studies that used questionnaires used a Likert scale for responses. Half of them were composed of five variation degrees [12,16] and the other half with seven variation degrees [13,18]. Furthermore, the use of questionnaires was heterogeneous in relation to discussed studies. This shows that there is no standardization. It is possible that evaluations that used Likert scales should vary according to the application under study, but currently there is not specific research about this topic.

No author proposed new approaches for evaluation methods. Some authors suggested and used some instruments, such as the CSUQ questionnaire, MBTI questionnaire, Folding Tests, Eyes Test, Fugl-Meyer Assessment, PAES, the think-aloud method, and modified questionnaires from Boot et al [23] and Brooke [25]. However, it is clear that there is still no protocol for interface usability evaluation for serious games, especially for specific populations such as elderly.

### Usability Evaluation Methods Used in Selected Studies

Regarding usability evaluation methods of this review, we observed a variety of techniques applied by authors in different approaches, with low adherence between selected studies. Table 4 summarizes these methods and their intended use in related work.

This heterogeneity shows that researchers are concerned with particularities of their samples and their experiments, avoiding biases. This situation also shows a lack of standardization, at least partially, of a protocol or tools for evaluating games based on gestures and/or movements. This makes the choice of best methods or techniques difficult for a unified approach in future work.

The benefits of this diversity are a good number of approaches that used evaluation instruments are well established in the literature. A group of authors, for instance, applied tools to evaluate cognitive, emotional, and motor skills in experiments. Another group applied methods and techniques to evaluate user satisfaction, user perception, and user performance.

It is important to keep it in mind during the creation of a standardization process to evaluate usability because there are consolidated techniques and scales for measuring gains according to the specificity of each approach. For example, comparison of games for people with upper limb impairment can use the Fugl-Meyer test as part of the evaluation process. In a rehabilitation context, it can support decisions and appropriately choose and validate a game according to the user’s profile.

Considering the evaluation instruments used by selected studies, it is possible to think about advantages and disadvantages to define the basis of a standardized procedure to evaluate usability of gesture-based games regardless of the user experience.

In reference to sociodemographic and health questionnaires, it is important to verify the age and previous game experience (software and hardware) of the user. This can help divide the user groups and the test conduction. Preliminarily, physical and cognitive limitations can also be identified to avoid health hazards and contribute to a better gaming experience.

Interviews are recommended when the number of participants is small, given that the collection data are qualitative and demands a time-consuming analysis. For example, in preliminary assessments, groups are smaller and interviews are useful to understand the reasoning of the user facing a problem. On the other hand, the subjective nature of the interviews leads to different interpretations by the evaluators in groups with many users.

Observations also require time for analysis because of the large amount of data acquired. However, it may provide a different perspective to the evaluator that other techniques do not provide, such as the moment when a problem occurred. Used together with video recording, observations can facilitate the review process of user actions and enrich the usability evaluation.

Think-aloud protocols affect user performance because they force the participant to do more than one task at a time, resulting in the loss of focus on game tasks or in unintended actions. Another problem is that motion-based systems use sensors for speech recognition as an interaction technique (because the feature is available on motion-sensing input devices), making it impossible in the use of this protocol. Therefore, we not recommend it in a usability evaluation for motion-based games.

User performance reporting is an interesting instrument because the game software can collect the measures during the interaction process and it is useful for collating with other assessment tools. For example, you can make a relationship between time spent executing the task with acceptance of the technology in order to see whether the user liked the game or not. However, if analyzed without comparison with another instrument, it is essential to instruct users in a very specific way about how they should perform the task in order to obtain balanced results. This is important because there is an implicit relationship between task performance metrics, such as speed and accuracy. The participant may be faster, but be less accurate, or the participant can increase accuracy, but decrease the speed [21].

**Table 4:** Purposes of each method in selected studies.

Method and study ID		Used to...
**Sociodemographic and health questionnaire**		
	Komlódi et al [11]	Identify the users’ profiles
	Harrington et al [18]	Identify the users’ profiles and their physical limitations
	Nakai et al [19]	Identify the users’ profiles
	Sheu et al [20]	Obtain personal information
**Author’s questionnaires**		
	Legouverneur et al [12]	Get the user satisfaction and verify if familiarization with the games have influence on user preference measures
	Norouzi-Gheidari et al [14]	Evaluate the acceptance of virtual reality technology for games
	Shin et al [16]	Test the usability of the game from expert perspective
	Harrington et al [18]	Evaluate the user task performance and user satisfaction
	Nakai et al [19]	Get the user feedback about the game
**Observation**		
	Komlódi et al [11]	Verify the users’ behaviors and issues during a session
	Shin et al [16]	Assess the usability and the negative effects of the game
**Video recording**		
	Komlódi et al [11]	Verify the task time and get usability issues
	Legouverneur et al [12]	Used to elicit users’ behavior and performance
	Nakai et al [19]	Verify the users’ behaviors during a session
**User performance report**		
	Norouzi-Gheidari et al [14]	Evaluate success rate, speed, and accuracy during the tasks
**Think-aloud protocol**		
	Nakai et al [19]	Verify the users’ behaviors during a session
**Interview**		
	Liu et al [15]	Collect suggestions about the game in open questions (qualitative data analysis)
	Harrington et al [18]	Identify what users liked or not in each game, the reason of the answers, their frustration, and what form of aid is the most beneficial
	Sheu et al [20]	Verify the user experience and get doubts
	Fang et al [17]	Evaluate the combined use of exercises and verify the user experience
**Other relevant tools**		
	Komlódi et al [11]	Verify cognitive, motor, and emotional aspects (MBTI^a^, Folding Test, Eyes Test)
	Francese et al [13]	Measure the user satisfaction during interaction process, the usability and the quality of the system, and the presence and immersion (ASQ^b^, CSUQ^c^, Presence Questionnaire)
	Norouzi-Gheidari et al [14]	Assess of sensorimotor function of upper limbs (Fugl-Meyer Assessment)
	Shin et al [16]	Assess of sensorimotor function of upper limbs, and functional capacity (Fugl-Meyer Assessment, Barthel)
	Fang et al [17]	Ensure that participants were physically and mentally ready to perform to play, and check the degree of pleasure during the tasks (PARQ^d^, PAES^e^)
	Harrington et al [18]	Evaluate gameplay experience, check the acceptance of technology, the familiarity with technology, and measure the usability (effectiveness, efficiency and satisfaction) (Technology Experience and Videogame Experience questionnaires, TAM^f^, SUS^g^)
	Sheu et al [20]	Ensure that participants were physically and mentally ready to perform the tasks, check the degree of pleasure during the activities, and measure the usability (effectiveness, efficiency and satisfaction) (PARQ, PAES, SUS)

^a^MBTI: Myers-Briggs Type Indicator.

^b^ASQ: After-Scenario Questionnaire.

^c^CSUQ: Computer System Usability Questionnaire.

^d^PARQ: Physical Activity Readiness Questionnaire.

^e^ PAES: Physical Activity Enjoyment Scale.

^f^TAM: Technology Acceptance Model.

^g^SUS: System Usability Scale.

Finally, the use of posttest questionnaires without validation is not a good practice in evaluations because it is not certain that the listed issues are relevant and appropriate inside the application context. Moreover, it is necessary to manage specific questionnaires at the end of experimental session because they can tire the participants leading to them not adequately indicating their impressions about the system. On the other hand, it is important to create specific questionnaires to evaluate the usability of motion-based games. In this case, the questionnaires must be validated in preliminary studies to prove the quality of the results. Preferably, they must be applied with other posttest tools in preliminary assessments in order to define specific time for the experiment.

### Suggested Guidelines for a Usability Evaluation Approach

In view of our impressions, a suggested usability evaluation approach for gesture-based and motion-based games can follow some guidelines within the pretest, test, and posttest stages.

In the pretest stage, we recommend the use of instruments capable of distinguishing users with previous experience considering the resources under evaluation and capable of indicating user limitations that may interfere during the experiment. We suggest the use of a questionnaire to characterize the sample. This instrument must track cognitive and physical aspects to avoid biases. For example, it can identify a problem that affects the understanding of the game activities during the sessions, or even a limitation that affects the movement required in gestural interaction. Among the instruments listed in the studies included in this systematic review were PARQ and PAES.

During the test, we suggest collecting performance and physiological user data using software. User performance reporting is useful if the purpose is to verify the user’s progress during the interaction process, usually from objective measures, such as speed and accuracy. However, it is interesting to use tracker video software to study the evolution of movements. Physiological measures can also be useful to identify emotions that may affect the user’s interaction with the game tasks. Heart rate, for example, can show evidence of stress during the interaction process. Observations can also record the evaluator’s perception during the session. We also recommend testing different versions of the same game to identify relevant issues, such as a version that uses a specific guideline and another without using it.

In the posttest stage, one can opt for a qualitative or quantitative analysis, depending on the purpose of the evaluation. If the option is for qualitative results, we recommend an interview to collect the user’s perception of the game. On the other hand, in a quantitative analysis, the use of instruments such as TAM and SUS, for example, may be useful for evaluating user acceptance and user satisfaction. In this case, we recommend scales that allow for statistical analysis, such as a Likert scale.

### Conclusions

Results show that there is no standardization in evaluation methods because they use different analysis variables. The definition of usability in games, especially in relation to gestural interaction, and of who should be evaluated and how it should be assessed, were not evident in the selected studies. Some studies evaluated users and others experts, using qualitative and/or quantitative methods.

We observed that the studies in this systematic review do not use the same methods in the user selection process or similar criteria in pilot tests or protocols for usability evaluations. There was also not similarity between the questionnaires and answer options.

We suspect that the lack of studies and methods (as well as theoretical foundation) that indicate the appropriate interaction techniques for each experience or application is the reason for the lack of standardization.

For this reason, the use of a standard usability evaluation process for gesture-based games and definition of criteria to enable a quantitative analysis in evaluations can be significant to this area. Therefore, understanding the different types of goals in usability evaluation and its implementation becomes relevant. Once the different types of goals have been established, it becomes possible, for example, to create a usability evaluation tool for rehabilitation of people with some type of motor or cognitive impairment, or even for unimpaired people with a focus on entertainment or training.

An evaluation for gesture-based games may also consider other factors in order to contribute to the usability applications. Physiological metrics, user anxiety levels, and stress issues are measures that could be collected during the evaluation. It is essential to consider these questions to advance the study in this area, defining at least one basic usability method to guide user assessments.

With this in mind, it is important to consider the adoption of a standard for usability evaluations for two reasons: to guide the validation of gesture-based and/or motion-based systems in future case studies and to check whether a particular hardware or software is suitable for their intended use. This valuation approach is substantial because the market regularly offers new interaction methods without consideration for how the public will use these solutions. As an example, the elderly, a group that is increasing globally, are using interaction systems more and more every day.

### Future Work

Our proposal is to validate the suggested approach in a usability evaluation protocol using, as a case study, two versions of a serious game based on gestures and movements (2D and 3D) for the elderly. We will regard three stages suggested in this study. We believe that this validation can be the basis to consolidate a standardized usability evaluation approach for gesture-based games based on our experiences and the identified studies in this review.

The focus for the elderly is justified by the fact that the game industry has been investing in this age group due to the growth of this population and increased life expectancy in most countries. In this systematic review, it was observed that some studies also used this population [12,17,18,20], with serious games for physical exercise and rehabilitation processes.

Adults today have greater contact with technologies and, therefore, will tend to use them more in the future. Easy access to a larger set of gestural interaction devices and general information systems will also contribute to the use of these technologies by a greater number of users.

## Abbreviations

ACM: Association for Computing Machinery
ASQ: After-Scenario Questionnaire
CSUQ: Computer System Usability Questionnaire
EFS: Evergreen Fitness System
MBTI: Myers-Briggs Type Indicator
PAES: Physical Activity Enjoyment Scale
PARQ: Physical Activity Readiness Questionnaire
SUS: System Usability Scale
TAM: Technology Acceptance Model
